# Deciphering the contribution of 5’-tiRNA- Lys-CTT as an emerging early diagnostic biomarker for hepatocellular carcinoma

**DOI:** 10.1007/s12672-026-04636-7

**Published:** 2026-02-13

**Authors:** Abinash Nayak

**Affiliations:** Department of Zoology, Utkal University Vani Vihar, Vani Vihar, Bhubaneswar, Odisha 751004 India

**Keywords:** Hepatocellular carcinoma, tRNA-derived small RNAs, 5’-tiRNA-Lys-CTT, Biomarker, Early detection, Precision oncology

## Abstract

Hepatocellular carcinoma (HCC) remains a leading cause of cancer mortality worldwide, with early detection critical for improving survival rates. Late-stage diagnosis contributes to dismal prognosis. Current surveillance relies on ultrasonography and serum alpha-fetoprotein (AFP), yet AFP exhibits limited sensitivity and specificity for early HCC. Emerging non-coding RNAs, particularly tRNA-derived small RNAs (tsRNAs), offer stable, detectable alternatives for liquid biopsy. Among these, 5′-tiRNA-Lys-CTT, a 5′-tRNA half derived from tRNA-Lys-CTT, has shown promise. This commentary explores the potential of 5′-tiRNA-Lys-CTT, a tRNA-derived small RNA (tsRNA), as an emerging biomarker for early HCC detection. It integrates biology, diagnostic performance along with recent advancements in tsRNA research for Hepatocellular carcinoma (HCC), and discusses potential clinical applications for enhanced early detection and personalized therapy. Ultimately, 5′-tiRNA-Lys-CTT represents a promising avenue for liquid biopsy-based screening, potentially revolutionizing HCC management.

## Introduction

Hepatocellular carcinoma (HCC) accounts for the sixth highest incidence of cancers as well as the third leading cause of cancer deaths with over 900,000 new cases reported every year with a five-year survival rate of below 20% for late stages [1]. Early diagnosis of HCC is essential as treatments such as resection or ablation are associated with a survival rate greater than 70% for Barcelona Clinic Liver Cancer (BCLC) 0/A stages [2]. Present surveillance is based on biannual ultrasound with or without alpha-fetoprotein (AFP), but sensitivity limitations (47–63% for HCC at an early stage) and specificity, particularly in cirrhosis, highlight the necessity for new biomarkers [3–5].

Non-coding RNAs, such as microRNAs and long non-coding RNAs, have been identified as promising HCC biomarkers, but tRNA-derived small RNAs (tsRNAs)—14–40 nucleotide-length fragments of tRNA cleavage—are an underinvestigated frontier [6, 7]. Formed under stress by means of angiogenin among other enzymes, tsRNAs modulate gene expression, translation, and cellular functions, with dysregulation being involved in oncogenesis [8].

The study by Yuan et al. moves this field forward by proposing that 5′-tiRNA-Lys-CTT (a 5′-tRNA halve of tRNA-Lys-CTT) can be a promising early HCC biomarker [9]. Using high-throughput sequencing on BCLC 0/A-stage HCC tissues, they screened 316 dysregulated tsRNAs, selecting 5′-tiRNA-Lys-CTT for validation due to its upregulation. Validation employed qRT-PCR with specific primers for 5′-tiRNA-Lys-CTT, normalized to U6 or cel-miR-39 spike-in, in independent cohorts (50 paired tissues from histologically confirmed HCC patients without prior treatment, 110 serum samples from HCC vs. controls with matched chronic liver disease, and 5 HCC cell lines). Sample selection criteria included BCLC 0/A staging and no confounding treatments. ROC curves, sensitivity/specificity, and statistical tests (Mann-Whitney U, *p* < 0.001) confirmed upregulation. Stability tests (agarose electrophoresis, Sanger sequencing) confirmed its serum detectability [9]. Present surveillance is based on biannual ultrasound with or without alpha-fetoprotein (AFP), but sensitivity limitations (47–63% for HCC at an early stage) and specificity, particularly in cirrhosis, highlight the necessity for new biomarkers. Specifically, the study by Wang et al. highlights the predictive value of tumor compression of the hepatic or portal vein in HCC, which underscores the need for more accurate biomarkers. Additionally, imaging features such as tumor compression of the hepatic or portal vein predict advanced disease progression, further underscoring the need for more accurate, non-invasive biomarkers [10].

Current surveillance relies on abdominal ultrasonography (US) combined with serum AFP levels, recommended every 6 months for at-risk populations. However, US is operator-dependent and less effective in obese patients or those with cirrhosis, while AFP has suboptimal sensitivity (40–65% for early-stage HCC) and specificity (80–94%), leading to frequent false positives/negatives [11]. Recent advances in molecular biology have shifted focus toward novel biomarkers, including non-coding RNAs, circulating tumor DNA (ctDNA), and extracellular vesicles (EVs) [12]. Among these, tRNA-derived small RNAs (tsRNAs), particularly tRNA halves (tiRNAs), have emerged as stable, detectable entities in biofluids, offering potential for non-invasive “liquid biopsy” diagnostics. This commentary examines the role of 5′-tiRNA-Lys-CTT, a specific 5′-tiRNA derived from tRNA-Lys-CTT, in advancing HCC early detection.

## Biology and discovery of tiRNAs in cancer

tiRNAs are 30–40 nucleotide fragments generated from mature tRNAs under stress conditions via angiogenin-mediated cleavage. They regulate gene expression by inhibiting translation, modulating ribosome biogenesis, or interacting with RNA-binding proteins. In cancer, tiRNAs contribute to oncogenesis by promoting cell proliferation, migration, and metastasis. High-throughput sequencing has revealed dysregulated tiRNAs in various malignancies, including breast, lung, and prostate cancers, where they serve as diagnostic or prognostic indicators [9]. In HCC, tsRNA profiling identified differential expression in early-stage tissues. Specifically, 5′-tiRNA-Lys-CTT, a 5′-half from tRNA-Lys-CTT, was upregulated in tumor tissues, serum, and cell lines. Its stability in biofluids—resistant to RNase degradation—makes it ideal for liquid biopsy [9].

tRNA-Lys-CTT is a lysine tRNA isoform with anticodon CTT. Under cellular stress, angiogenin cleaves it at the anticodon loop, producing the 5′-tiRNA-Lys-CTT half (approximately 30–35 nt). This fragment exhibits robust secondary structure, conferring high stability and RNase resistance in biofluids, ideal for liquid biopsy [9]. Downstream targets include mRNAs in metabolic pathways (e.g., lipid metabolism, glycolysis), cancer pathways, and HCC-specific signaling (e.g., PI3K/AKT, Wnt). Bioinformatics predicts interactions with RNA-binding proteins or translation modulation. Functional assays show oncogenic activity: overexpression enhances HCC cell proliferation (CCK-8, colony formation, EdU) and migration (Transwell), while knockdown suppresses these phenotypes [8]. Associations with HCC etiologies include strong correlation with cirrhosis (common in viral and non-viral HCC); preliminary data suggest relevance across HBV/HCV, NASH, though etiology-stratified studies are limited [13].

## Recent developments in tsRNA research for HCC

Recent advances in tsRNA research in HCC Post-2024, tsRNA research has picked up speed, disclosing their pathogenic roles in HCC and clinical relevance. In a 2025 report, tsr_019759 (5′-tiRNA from tRNA-Val-AAC) was found to be upregulated in HCC tissues and serum and associated with poor prognosis (tumor grade, size, TNM stage) [13]. It facilitates tumorigenesis through TNFSF15/JAK2/STAT3 inhibition and M2 macrophage polarization and the creation of an immunosuppressive microenvironment. Cell membrane-modified nanoparticles targeting tsr_019759 suppressed HCC tumor growth in xenografts, opening up therapeutic options [13].

Emerging developments in non-coding RNA biology have deepened the horizon of circulatory biomarkers for HCC. tsRNA expression profiles have been reported to distinguish HCC from cirrhosis and chronic hepatitis B [14]. Multi-omics strategies combining cfDNA methylation, exosomal RNAs, and proteomics have demonstrated high diagnostic specificity [15]. Machine learning–based multimarker models now integrate biochemical markers with transcriptomic signatures to increase surveillance accuracy [16]. These advances suggest a move toward integrative biomarker panels over single-analyte approaches to early HCC detection.

Another 2025 study evaluated the value of hsa_tsr014055 (tsRNA-Val-5-0035) as a blood biomarker with an AUC of 0.79 for individual factors and 0.89 for the AFP/DCP blends for diagnosis of HCC [17]. It is linked with the TNM stage, differentiation, and invasiveness, significantly decreased post-surgery, hinting towards its surveillance role. Enriched with the pathways of Hedgehog/cAMP, focussing on its mechanistic associations [18] Another inclusive 2025 analysis on the role of tsRNAs in liver disorders revealed 13 upregulated and 4 downregulated tsRNAs, causing the progression of MASLD to HCC [19]. The potential for a biomarker comes from fluid stability and detectability [19].

Previous results include METTL1-mediated m7G modification of tRNAs, which enhance the malignant potential of HCC under stress conditions, and 3′tsRNA-LeuCAG that supports ribosome biogenesis/apoptosis resistance [20]. The tRF-Gln-TTG-006 tRF has an AUC of 0.86 for early HCC detection [16]. Integration involving AI-radiomics analysis and tsRNAs has AUC values above 0.80 [21]. Combinations with ctDNA or microbiome markers improve early detection [22].To contextualize, Table 1 compares recent tsRNA biomarkers in HCC, highlighting their diagnostic and prognostic potential.

**Table 1:** Comparison of diagnostic performance of tsRNA biomarkers for early-stage HCC

Biomarker	Study (year)	Sample Type	AUC for Early HCC	Sensitivity (%)	Specificity (%)	Key advantages to AFP/DCP	References
5′-tiRNA-Lys-CTT	Yuan et al. (2025)	Serum/Tissue	0.764	72.5	75.0	Higher early-stage sensitivity/specificity; RNase-resistant stability for liquid biopsy; fewer confounders from benign liver disease (vs. AFP elevated in cirrhosis/hepatitis); better discrimination in validation cohorts	[9]
tRF-Gln-TTG (mitochondrial)	Zhan et al. (2024)	Serum	0.890	85.0	82.0	Variable stability	[23]
tiRNA-5′-Val	Mo et al. (2019, updated 2024)	Serum	0.820	78.0	80.0	Stable but assays not standardized	[24]
tsRNA Panel (5 tsRNAs)	Li et al. (2021)	Plasma	0.850	80.0	77.0	More stable	[25]
tRF-3b	Yang et al. (2022)	Serum	0.810	76.0	79.0	Less prone to degradation	[26]

## Clinical translation potential of 5′-tiRNA-Lys-CTT

The clinical translation of 5′-tiRNA-Lys-CTT as a biomarker for hepatocellular carcinoma (HCC) represents a promising advancement in addressing the critical need for improved early detection and surveillance. Traditional methods—biannual ultrasonography ± alpha-fetoprotein (AFP)—offer only 40–65% sensitivity for early-stage (BCLC 0/A) disease and are often confounded by cirrhosis or hepatitis, leading to missed small tumors or false positives. This 5′-tRNA-derived half exhibits excellent stability in serum due to RNase resistance, enabling reliable qPCR detection in liquid biopsy samples. High-throughput sequencing and validation in cohorts (50 tissues, 110 serum samples, HCC cell lines) confirmed significant upregulation in early HCC, with strong correlations to tumor size, BCLC stage, and cirrhosis. ROC analysis showed superior diagnostic performance compared to AFP and DCP (PIVKA-II), with even higher AUC when combined into multi-marker panels [9].

Despite promising findings, research on 5′-tiRNA-Lys-CTT as an HCC biomarker remains in its early stages and faces several important limitations that must be addressed before widespread clinical adoption [11]. Detection methods, while effective (qPCR with normalization to U6 or spike-ins), lack standardized protocols across laboratories. Variations in primer design, RNA extraction techniques, normalization strategies, and cutoff thresholds could affect reproducibility and comparability of results [12]. Furthermore, functional insights into downstream targets and oncogenic mechanisms remain preliminary, relying heavily on bioinformatics predictions and in vitro assays without extensive in vivo validation or mechanistic dissection (e.g., direct RNA–protein or RNA–RNA interactions) [20]. Longitudinal data are absent, so the biomarker’s utility for monitoring treatment response, recurrence, or prognosis is unproven. No large-scale, multicenter, prospective studies have been conducted, and phase II/III biomarker validation trials are lacking [23]. These gaps highlight the need for broader, more robust validation to confirm diagnostic performance, establish clinical cutoffs, and evaluate cost-effectiveness in real-world surveillance settings.

## Conclusion

The finding of 5′-tiRNA-Lys-CTT as a serum-detectable, stable, and functionally active small RNA that is elevated in early-stage HCC is a significant and novel development. The current data indicates its potential as an LBB biomarker, especially when combined with well-established ones. However, rigorous analytical standardization, independent validation across diverse cohorts, well-designed prospective surveillance studies and mechanistic work are essential to clarify its true clinical utility. If these steps are successful, 5′-tiRNA-Lys-CTT could make significant contributions to improve early detection and subsequent improved prognosis for patients with threats of developing HCC. Its integration with the new-generation techniques has the promising ability to bring revolutionary outcomes in HCC. The discovery reinforces the paradigm shift towards tsRNAs as indispensable components of the non-coding transcriptome and secures a bright future for this molecular class in non-invasive cancer diagnostics.

## Future directions

Future research should focus on multicentric validation of 5’-tiRNA-Lys-CTT across diverse etiological backgrounds such as HBV, HCV, and NASH-related HCC. Prospective, multicenter validation of 5’-tiRNA-Lys-CTT is essential, incorporating ctDNA and AI for dynamic models. Additionally, mechanistic validation of predicted mRNA targets through luciferase reporter assays or pull-down experiments are necessary to confirm direct interactions. Integration of 5’-tiRNA-Lys-CTT with other established biomarkers such as GP73, cfDNA methylation markers, and imaging data may yield clinically applicable multimodal models. Exploring tsRNA therapeutics, like nanoparticle inhibitors, could personalize HCC management. Addressing global disparities in surveillance access remains key. Future research should focus on Prospective multicenter cohorts (diverse etiologies/ethnicities), etiology-specific validation, multi-omics integration (e.g., with ctDNA mutations/methylation panels for > 90–95% accuracy), AI-driven models combining 5′-tiRNA-Lys-CTT with existing panels (GALAD, HES), EV enrichment for point-of-care testing, therapeutic targeting of pathways (e.g., synergy with PD-1 inhibitors). Addressing these things could transform early HCC detection into a more precise, accessible, and patient-tailored diagnostic paradigm.

## Data Availability

No datasets were generated or analysed during the current study.
